# Characteristics of Myeloid Differentiation and Maturation Pathway Derived from Human Hematopoietic Stem Cells Exposed to Different Linear Energy Transfer Radiation Types

**DOI:** 10.1371/journal.pone.0059385

**Published:** 2013-03-12

**Authors:** Satoru Monzen, Hironori Yoshino, Kiyomi Kasai-Eguchi, Ikuo Kashiwakura

**Affiliations:** 1 Department of Radiological Life Sciences, Hirosaki University Graduate School of Health Sciences, Hirosaki, Aomori, Japan; 2 Radiation Effect Mechanisms Research Group, Research Center for Radiation Protection, National Institute of Radiological Sciences, Chiba, Japan; Kagoshima University Graduate School of Medical and Dental Sciences, Japan

## Abstract

Exposure of hematopoietic stem/progenitor cells (HSPCs) to ionizing radiation causes a marked suppression of mature functional blood cell production in a linear energy transfer (LET)- and/or dose-dependent manner. However, little information about LET effects on the proliferation and differentiation of HSPCs has been reported. With the aim of characterizing the effects of different types of LET radiations on human myeloid hematopoiesis, *in vitro* hematopoiesis in Human CD34^+^ cells exposed to carbon-ion beams or X-rays was compared. Highly purified CD34^+^ cells exposed to each form of radiation were plated onto semi-solid culture for a myeloid progenitor assay. The surviving fractions of total myeloid progenitors, colony-forming cells (CFC), exposed to carbon-ion beams were significantly lower than of those exposed to X-rays, indicating that CFCs are more sensitive to carbon-ion beams (*D*_0_ = 0.65) than to X-rays (*D*_0_ = 1.07). Similar sensitivities were observed in granulocyte-macrophage and erythroid progenitors, respectively. However, the sensitivities of mixed-type progenitors to both radiation types were similar.

In liquid culture for 14 days, no significant difference in total numbers of mononuclear cells was observed between non-irradiated control culture and cells exposed to 0.5 Gy X-rays, whereas 0.5 Gy carbon-ion beams suppressed cell proliferation to 4.9% of the control, a level similar to that for cells exposed to 1.5 Gy X-rays. Cell surface antigens associated with terminal maturation, such as CD13, CD14, and CD15, on harvest from the culture of X-ray-exposed cells were almost the same as those from the non-irradiated control culture. X-rays increased the CD235a^+^ erythroid-related fraction, whereas carbon-ion beams increased the CD34^+^CD38^−^ primitive cell fraction and the CD13^+^CD14^+/−^CD15^−^ fraction. These results suggest that carbon-ion beams inflict severe damage on the clonal growth of myeloid HSPCs, although the intensity of cell surface antigen expression by mature myeloid cells derived from HSPCs exposed to each type of radiation was similar to that by controls.

## Introduction

Hematopoietic stem and progenitor cells (HSPCs) have high proliferative potential and mature into functional blood cells [1]. The highly glycosylated transmembrane protein CD34 is strongly expressed on HSPCs, and CD34^+^ cells capable of reconstituting hematopoiesis are found at low frequency in mononuclear cells of peripheral blood (<0.5%) and bone marrow (1–3%) [2], [3]. HSPCs without some growth factors is the quiescent cells [4], and classified to the lineage of common lymphoid progenitor, common myeloid progenitor, granulocyte/monocyte progenitors and megakaryocyte/erythrocyte progenitors (MEP) by cell surface antigens with CD34 [5]. This regenerative system is consequently extremely sensitive to extracellular oxidative stress such as that inflicted by ionizing radiation and chemotherapeutic agents [6]–[8]. Ionizing radiation is generally classified into high- and low-linear energy transfer (LET) radiation according to its physical effects. The main biological effect of high-LET radiation is direct action on DNA itself, whereas low-LET radiation induces indirect cellular damage by free radicals [9]–[11]. Thus, similar to high-LET radiation, carbon-ion beams exert a greater biological effect than electron beams or X-rays.

In clinical practice, high-LET radiation, such as proton beams and carbon-ion beams, is more effective in cancer therapy than low-LET radiation such as γ-rays or X-rays. Tobias *et al.* have reported that ion tracks in the cell nucleus differ according to the energy and type of carbon-ion beam [12]. However, little is known about the effect of carbon-ion beams on the proliferation and differentiation of human HSPCs. Elucidation of the effects of LET differences on normal tissue, particularly a high sensitivity hematopoietic system, will not only improve quality of life of cancer patients but also suggest a method for recovery from symptoms that emerge from radiation.

In previous studies, we demonstrated that CD34^+^ megakaryocytic progenitors, colony-forming unit megakaryocytes (CFU-Meg), are much more sensitive to carbon-ion beams than to X-rays and that carbon-ion beams affect megakaryocytopoiesis differentiation of human HSPCs at the gene expression level [13]–[15]. However, little has been reported about the differences between the biological effects of carbon-ion beams and X-rays on the proliferation and differentiation of myeloid progenitors, which commit to neutrophils, monocytes/macrophages, and erythrocytes. Observation of the expression of cell surface antigens with the aim of discriminating the differentiated lineages of hematopoietic cells is an effective way of assessing cell conditions following radiation exposure. Because ionizing radiation exposure is known to induce retardation of the cell cycle, we hypothesized that differentiation and proliferation ability of cells exposed to high-LET radiation will be lower than that of cells exposed to low-LET radiation. Although the suppression of cell proliferation of HSPCs exposed to ionizing radiation will suppress LET dependence, the fraction of matured cells uniquely varied with LET. In the present study, the biological characteristics of the myeloid differentiation and maturation pathway derived from human CD34^+^ HSPCs exposed to carbon-ion beams or X-rays were compared.

## Results

### Clonal growth of CD34^+^ HSPCs exposed to carbon-ion beams or X-rays

**Figure 1:**
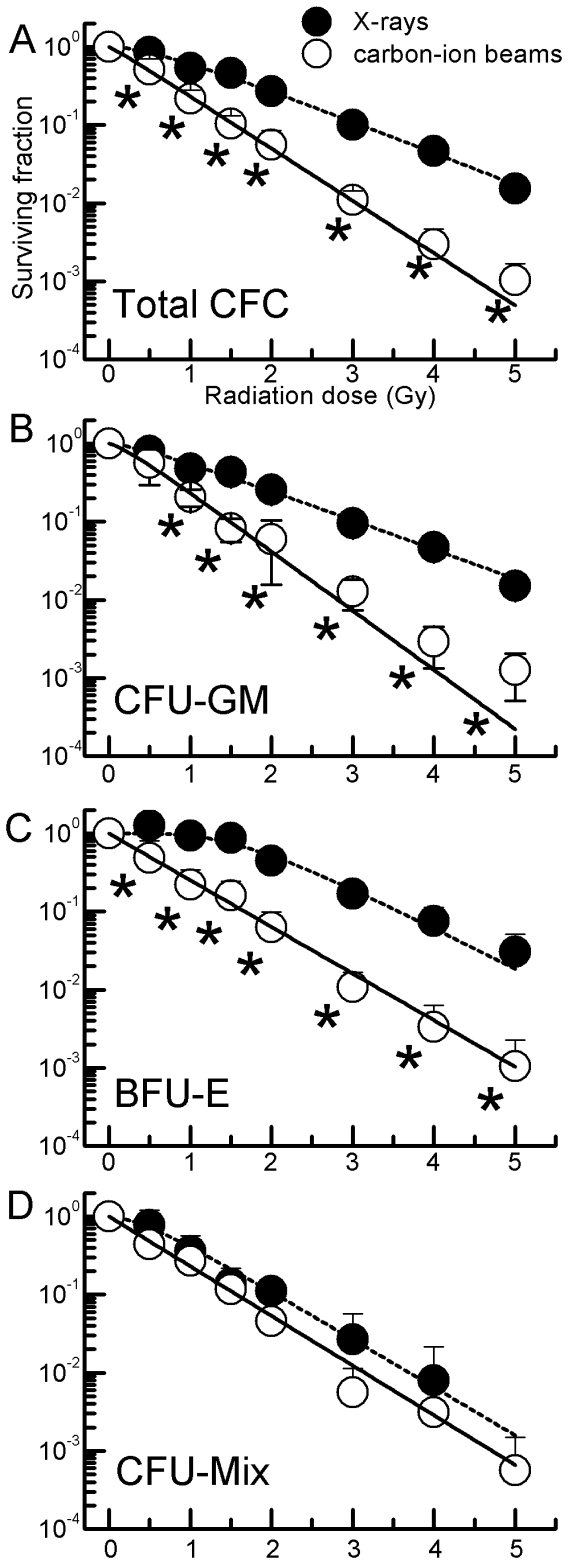
Radiation dose-response curves for CB CD34^+^ hematopotoietic progenitor cells. CD34^+^ cells were irradiated with 0.5–5.0 Gy X-rays (•) or carbon-ion beams (○) and assayed for the surviving fraction of total CFC [A], CFU-GM [B], BFU-E [C], and CFU-Mix [D] using methylcellulose cultures for 14 days. Values are the mean±S.E. of 4–6 separate experiments, performed in three wells. Curves were fitted as described in Materials and Methods. *P<0.05 vs. each X-ray dose.

Freshly prepared placental/umbilical cord blood (CB) CD34^+^ cells were exposed to 0.5–5.0-Gy carbon-ion beams or X-rays and were plated in methylcellulose semi-solid culture supplemented with an optimal cytokine combination. The radiation survival curves of total hematopoietic progenitors, colony-forming cells (CFCs), containing colony-forming unit granulocyte-macrophages (CFU-GM), erythroid burst-forming units (BFU-E), and granulocyte-erythroid-megakaryocyte-macrophage colony-forming units (CFU-Mix) are shown in Fig. 1. The parameters *D*_0_ and *n* characterizing radiosensitivity, obtained from these curves, are summarized in Table 1. The surviving fractions of CFCs exposed to carbon-ion beams were significantly lower in abundance than those of CFCs exposed to X-rays (Figure 1A), indicating that each progenitor is more sensitive to carbon-ion beams than to X-rays (*D_0_*: *P* = 1.40×10^−9^, *n*: *P* = 1.16×10^−7^ by ANOVA, Table 1). Moreover, *D_0_* and *n* for each progenitor after irradiation with X-rays and carbon-ion beams showed different values (*D_0_*: *P* = 1.75×10^−3^, *n*: *P* = 4.53×10^−8^ by ANOVA, Table 1). For BFU-E, the *n* values were significantly higher than those for other CFCs after irradiation with X-rays, whereas those after irradiation with carbon-ion beams were not significantly higher (Table 1). In contrast, for CFU-Mix, similar sensitivities were observed between X-rays and carbon-ion beams (Figure 1D and Table 1). The RBE_10%_ (comparison of 10% surviving fraction for carbon-ion beams/X-rays) of CFU-GM, BFU-E, CFU-Mix, and total CFC were 2.07, 2.12, 1.31, and 1.94, respectively, indicating that the biological effects of carbon-ion beams on human myeloid progenitors except for CFU-Mix are almost twice those of X-rays.

**Table 1:** Radiosensitivity of hematopoietic myeloid progenitor cells.

		CFU-GM	BFU-E	CFU-Mix	Total CFC
X-rays	*D_0_*	1.15±0.05*^a,b^*	0.83±0.04*^c^*	0.71±0.06*^d^*	1.07±0.09
	*n*	1.46±0.20	7.72±1.00*^e,f,g^*	1.88±0.58	1.85±0.33
Carbon-ion beams	*D_0_*	0.57±0.02*^h^*	0.73±0.03*^i^*	0.64±0.05	0.65±0.03*^j^*
	*n*	1.37±0.28	1.00±0.26*^k^*	1.00±0.16	1.10±0.11*^l^*
RBE_10%_		2.07	2.12	1.31	1.94

Values are the mean±SE. of 6–8 separate experiments. *P* values were calculated using two-factor factorial ANOVA and Tukey–Kramer test. A significant difference was observed between *D_0_* and *n* for radiation types (*D_0_*: *P* = 1.40×10^−9^, *n*: *P* = 1.16×10^−7^) and for each progenitor (*D_0_*: *P* = 1.75×10^−3^, *n*: *P* = 4.53×10^−8^). *^a^P* = 4.97×10^−2^ vs. BFU-E, *^b^P* = 1.33×10^−4^ vs. CFU-Mix, *^c^P* = 6.55×10^−3^ vs. CFU-Mix, *^d^P* = 9.49×10^−4^ vs. CFC, *^e^P* = 2.87×10^−3^ vs. CFU-GM, *^f^P* = 4.01×10^−2^ vs. CFU-Mix, *^g^P* = 4.64×10^−3^ vs. CFC in X-rays. *^h^P* = 3.79×10^−6^, *^i^P* = 0.00196, *^j^P* = 6.51×10^−5^, *^k^P* = 2.34×10^−3^, *^l^P* = 2.50×10^−2^ compared with X-rays.

### Proliferation and differentiation of CD34^+^ cells exposed to carbon-ion beams or X-rays

CD34^+^ cells exposed to 0.5 or 1.5 Gy carbon-ion beams or X-rays were cultured in serum-free medium supplemented with an optimal cytokine combination and then harvested on day 14. As shown in Table 2, the control culture containing 1×10^3^ non-irradiated CD34^+^ cells increased to 5.37±1.54×10^6^ cells. A similar increase was observed in the culture of cells exposed to 0.5 Gy X-rays. However, 0.5-Gy carbon-ion beams resulted in a dramatic suppression of cell proliferation (2.73±0.60×10^5^ cells). At 1.5 Gy of intensity, the cell numbers were 9.82±1.75×10^4^ (carbon-ion beams) and 3.69±0.35×10^5^ (X-rays), indicating that 1.5 Gy-irradiated CD34^+^ cells experienced only slight proliferation compared with non-irradiated controls.

**Table 2:** The total number of mononuclear cells generated in liquid culture.

	0 Gy	0.5 Gy	1.5 Gy
X-rays	5.37±1.54×10^6^	5.59±0.74×10^6^	3.69±0.35×10^5*a,b*^
Carbon-ion beams	—	2.73±0.60×10^5*c*^	9.82±1.75×10^4*d*^

CD34^+^ cells were exposed to X-rays or carbon-ion beams and cultured in serum-free medium supplemented with cytokines. On day 14, cells harvested from each culture were counted. Each experiment was performed as an independent experiment. Values are the means±S.E. of more than four independent experiments. *^a^P* = 4.90×10^−2^ vs. non-irradiated control, *^b^P* = 7.42×10^−3^ vs. 0.5 Gy in X-ray, *^c^P* = 2.50×10^−5^ vs. non-irradiated control, *^d^P* = 1.85×10^−5^ vs. non-irradiated control.

**Figure 2:**
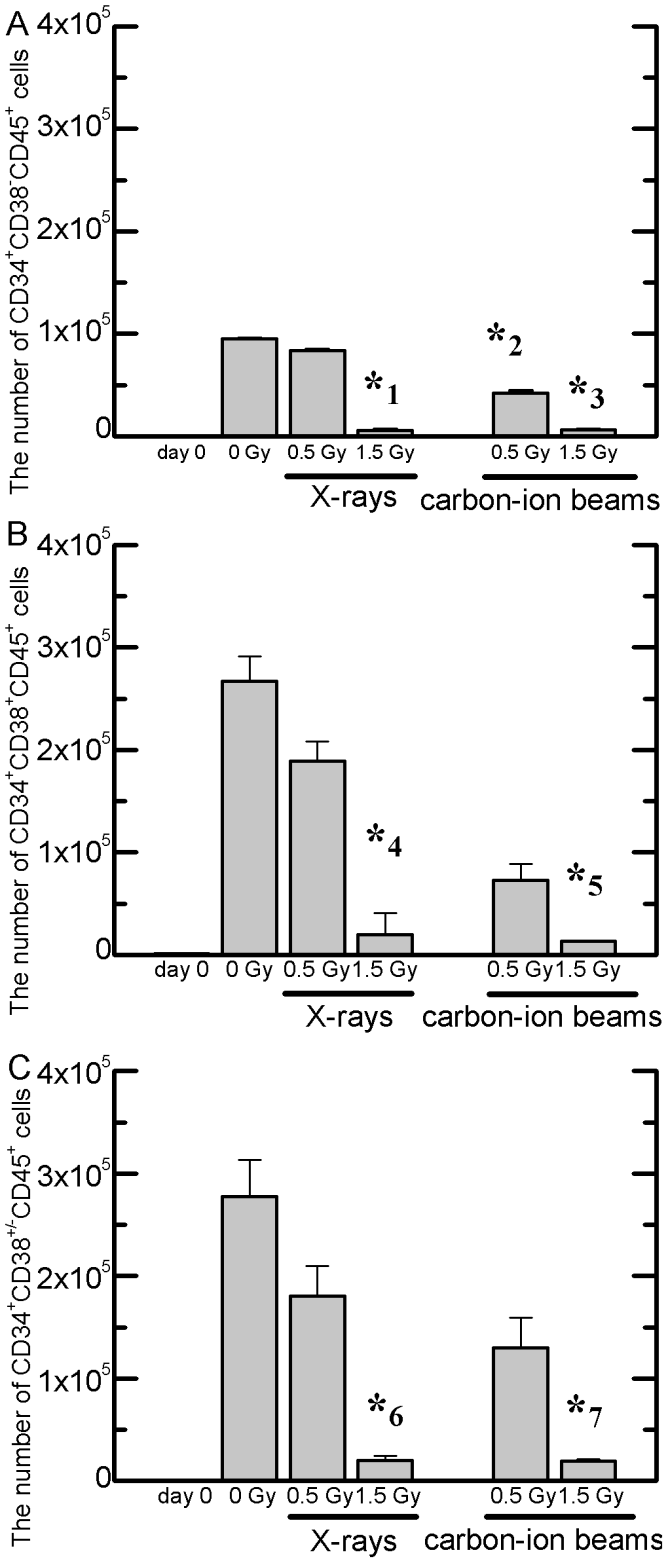
Responses of HSPCs to X-ray or carbon-ion beam irradiation. The numbers of CD34^+^CD38^−^CD45^+^ cells (panel A), CD34^+^CD38^+^CD45^+^ cells (panel B), and CD34^+^CD38^+/−^CD45^+^ cells (panel C) determined by flow cytometry were cultured until day 14 after irradiation. CD34^+^ cells were seeded in serum-free liquid cultures. Values are the means±S.E. of 6–8 separate experiments. * ^1^ *P* = 2.11×10^−15^, * ^2^ *P* = 1.52×10^−8^, * ^3^ *P* = 5.86×10^−16^, * ^4^ *P* = 4.46×10^−6^, * ^5^ *P* = 2.69×10^−6^, * ^6^ *P* = 8.88×10^−6^, * ^7^ *P* = 8.35×10^−6^ vs. non-irradiated controls on day 14.

Among the harvested cells described above, total number of primitive cells was evaluated based on the expression of early-stage hematopoiesis-related cell surface antigens. The CD34 antigen is a novel marker for human HSPCs and the CD45 antigen is a common leucocyte antigen. In addition, given that CD38 is a novel multifunctional ectoenzyme that is widely expressed in cells and tissues, most notably in leukocytes [16], CD34^+^CD38^−^ cells are more primitive cells than CD34^+^CD38^+^ cells. Accordingly we evaluated the expression of CD34, CD38, and CD45 antigens in the harvested cells. As shown in Figure 2, the total number of CD34^+^CD38^−^CD45^+^ immature HSPCs, CD34^+^CD38^+^CD45^+^ mature HSPCs, and CD34^+^CD38^+/−^CD45^+^ total HSPCs detected in the control culture increased dramatically by approximately 2,300-, 280-, and 280-fold, respectively. Each population observed in the culture of cells exposed to 0.5 Gy X-rays was at almost the same abundance as in the control; however, damage from the carbon-ion beam was observed only in primitive CD34^+^CD38^−^ CD45^+^ cells at 0.5 Gy (Fig. 2A). In addition, both the 1.5-Gy radiation types led to significant suppression of cell proliferation.

A phenotypic analysis of cell surface antigens in the terminal stages of myeloid differentiation was performed by flow cytometry (Table 3). The CD13 antigen is expressed on most cells of myeloid origin including neutrophils, eosinophils, basophils, and monocytes [17], [18]. CD14 is expressed mainly by macrophages and (at a 10-fold lower level) by neutrophils [19], [20]. The CD15 antigen is strongly expressed by neutrophils, eosinophils, and one of the monocytes. It is not expressed on normal erythrocytes, platelets, or lymphocytes [21]–[23]. CD235a (glycophorin A) is a well-defined major sialoglycoprotein in mature erythrocytes, reaching maximum expression at the early erythroblast stage and remaining at a constant number per cell throughout further differentiation [24], [25]. As shown in Table 2, although there was no significant difference in total cell numbers between the control and 0.5-Gy X-rays, the CD13^+^CD14^−/low^CD15^+^ eosinophil/neutrophil fraction observed in the latter was approximately half of that in the control, and a significant increase in the CD235a^+^ erythroid-related fraction was observed in the culture exposed to 0.5-Gy X-rays. The proliferation of cells exposed to 0.5-Gy radiation was much lower for carbon-ion beams than for X-rays, whereas the primitive populations-the CD34^+^CD38^−^CD45^+^ immature-HSPCs fraction (13.07%) and the CD34^+^CD38^+/−^CD45^+^ total HSPCs fraction (15.49%)-and the CD13^+^CD14^+/−^CD15^−^ basophile fraction were relatively more abundant compared with those in the control. In response to 1.5 Gy, although both cultures showed slight increases, almost all the cellular populations showed similar abundance except for the CD235a^+^ erythroid-related fraction.

**Table 3:** Responses of cell surface antigens to X-rays or carbon-ion beams.

Cell surface antigen		Day 0	Day 14				
				X-rays		Carbon-ion beams	
	Cell type		0 Gy	0.5 Gy	1.5 Gy	0.5 Gy	1.5 Gy
HSPCs (%)							
CD34^+^CD38^−^CD45^+^	immature	4.12±0.75	1.77±0.63	1.50±0.39	1.60±0.67	13.1±3.91^a^	5.63±1.13*^b^*
CD34^+^CD38^+^CD45^+^	mature	97.0±0.59	4.96±1.14	3.38±0.51	5.26±1.11	4.18±0.52	4.85±0.18
CD34^+^CD38^+/−^CD45^+^	total	98.1±1.11	5.16±0.54	3.23±0.56	5.48±0.76	15.5±2.83*^c^*	9.65±1.98*^d^*
Leukocytes (%)							
CD13^+^CD14^+^CD15^+/−^	monocytes, basophils	—	15.1±3.28	13.7±1.82	15.9±6.67	18.62±7.63	17.00±9.54
CD13^+^CD14^+/−^CD15^+^	neutrophils, eosinophils, one of the monocytes	—	15.1±2.48	11.3±2.77	11.9±3.34	17.42±6.49	20.1±9.41
CD13^+^CD14^+/−^CD15^−^	basophils	—	14.3±4.51	9.83±1.91	15.2±1.81*^e^*	34.3±5.78*^f^*	28.6±4.62*^g^*
CD13^+^CD14^−/lo^CD15^+^	eosinophils, neutrophils	—	17.2±3.60	7.71±0.98*^h^*	11.2±1.74*^i^*	14.8±4.93	14.3±4.99
CD13^+^CD14^+/−^CD15^+/−^	total	—	32.1±7.45	24.8±5.73	29.9±7.49	32.5±11.4	26.3±8.88
Erythrocytes (%)							
CD235a^+^	total	—	41.9±8.22	63.9±6.86*^j^*	64.1±5.43*^k^*	44.6±7.48	43.3±6.19

CD34^+^ cells exposed to carbon-ion beams or X-rays at indicated doses were seeded in serum-free liquid cultures. The percentage of each cell type among total MNCs was calculated using the compensated total MNCs. Values are means±S.E. (%) of more than four independent experiments. Each experiment was performed as an independent experiment.

a *P* = 1.73×10^−2^, *^b^P* = 1.46×10^−2^, *^c^P* = 4.72×10^−3^, *^d^P* = 4.37×10^−2^, *^e^P* = 4.99×10^−2^, *^f^P* = 1.72×10^−2^, *^g^P* = 4.11×10^−2^, *^h^P* = 1.91×10^−4^, *^i^P* = 4.68×10^−2^, *^j^P* = 4.36×10^−2^, *^k^P* = 4.16×10^−2^ vs. non-irradiated controls.

Considering only cell types that showed significant differences in the cell fraction of hemocytes, we performed linear regressions of radiation dose on expression of cell surface antigen and of radiation dose on number of each cell type after confirming the normality of the explanatory variables. For most cell types, the regressions were significant. In contrast, radiation dose was not correlated with expression of cell surface antigen (Table 4).

**Table 4:** Linear regression analysis for each cell fraction.

	*r* ^2^			
	dose vs. cell numbers		dose vs. expression	
	X-rays	^12^C-ion beams	X-rays	^12^C-ion beams
CD34^+^CD38^−^CD45^+^	0.333*^a^*	0.401*^b^*	0.001	0.01
CD34^+^CD38^+/−^CD45^+^	0.354*^c^*	0.474*^d^*	0.001	0.03
CD13^+^CD14^+/−^CD15^−^	0.306*^e^*	0.344*^f^*	0.01	0.10
CD13^+^CD14^−/lo^CD15^+^	0.143	0.372*^g^*	0.07	0.01
CD235a^+^	0.277*^h^*	0.414*^i^*	0.18	0.001

CD34^+^ cells exposed to carbon-ion beams or X-rays at indicated doses were seeded in serum-free liquid cultures. Cell fraction showing significant differences for X-rays or carbon-ion beams on day 14 were used for linear regression analysis. *^a^P* = 3.20×10^−2^, *^b^P* = 2.0×10^−3^, *^c^P* = 4.8×10^−2^, *^d^P* = 1.0×10^−4^, *^e^P* = 9.0×10^−3^, *^f^P* = 5.0×10^−3^, *^g^P* = 4.0×10^−3^, *^h^P* = 1.2×10^−2^, *^i^P* = 2.0×10^−3^.

## Discussion

In the present study, the biological characteristics of the myeloid differentiation and maturation pathway following exposure of human CD34^+^ cells to carbon-ion beams or X-rays were evaluated. No statistically significant difference in the parameters *D*_0_ and *n* characterizing the radiosensitivity was observed among the progenitors exposed to carbon-ion beams (Fig. 1 and Table 1), whereas significant differences were observed following exposure to X-rays; in particular, BFU-E showed lower radiosensitivity at 2 Gy. CFU-GM has high frequency in myeloid progenitors (data not shown) and low sensitivity to X-rays, whereas *D_0_* and *n* values of all CFCs irradiated with carbon-ion beams were similar. These results showed that the differentiation stage of human myeloid progenitors is not related to carbon-ion beam radiosensitivity, although we had not been able to predict this from our hypothesis. Our previous study reported that the radiosensitive parameters of CD34^+^ CFU-Meg obtained under optimal conditions with IL-3+SCF+TPO were *D_0_* = 0.71 Gy in carbon-ion beams and *D_0_* = 1.12 in X-rays [26]; the *D*_0_ values obtained in this study (0.65 and 1.07, respectively, for CFCs) are very similar to those reported previously (Table 1). In contrast, no significant difference was observed for CFU-Mix between the radiation types (Fig. 1 and Table 1). Although the present study does not fully explain this observation, CFU-Mix was a minor population (10%) in CFCs (data not shown) and CFU-Mix is known to be a relatively more primitive population than CFU-GM and BFU-E. Accordingly, these results suggest that the biological effects of X-rays depend on the differentiation stage in the hematopoietic pathway, in accordance with Bergonie–Tribondeau's law, whereas carbon-ion beams inflict nonspecific damage on HSPCs.

In the analysis of liquid culture, the total number of mononuclear cells generated in the culture of CD34^+^ cells exposed to 0.5-Gy X-rays was almost the same as that for the control; however, the CD13^+^CD14^−/low^CD15^+^ eosinophil/neutrophil fraction decreased, whereas the CD235a^+^ erythroid-related fraction increased. A similar tendency was observed with exposure to 1.5-Gy X-rays, although only few cells were generated in that culture. Our previous study finding that X-rays induced upregulation of several genes associated with the early stages of hematopoiesis, megakaryocytic maturation, and antioxidant systems suggests that ionizing radiation promotes both megakaryocytopoiesis and thrombopoiesis [15]. A similar promotion was confirmed in the myeloid differentiation and maturation pathway in the present study. In particular, the present results showed that X-rays promote mainly erythroid differentiation. Because erythropoiesis and megakaryocytopoiesis are derived from MEP of the same progenitor [27], it is possible that these specific promotions are the results of MEP activation by X-rays. In contrast, the cell proliferation observed in the culture of CD34^+^ cells following exposure to carbon-ion beams was lower than that following exposure to X-rays. The abundance of the primitive population of CD34^+^CD38^−^ cells was approximately 4–9-fold of that in the control, and CD34^+^CD45^+^ cell populations were also observed in higher abundance in the culture of the residual cells after exposure. Based on the present results, it is difficult to explain why carbon-ion beams induce immature HSC populations. However, our previous study demonstrated that the mRNA levels, that is generally expressed to HSPCs by stimulation of some growth factors for megakaryocytopoiesis [27], were upregulated in the early stage (FLI1, HO1, and NQO1) and maturation stage (Tie-2, CD62P, PECAM1, and CD44) with 0.5–2.0 Gy carbon-ion beam irradiation [13], [14]. In contrast, the expression variance of these same genes was not observed on X-ray irradiation conditions [15]. In addition, we have demonstrated normal terminal maturation of megakaryocytes in the residual surviving cells exposed to heavy-ion beam irradiation [14]. Therefore, though the residual surviving cells exposed to heavy-ion beams appeared to be normal cells and contained normal quiescent HSCs, further approaches will be required to determine the precise mechanisms underlying these issues.

Furthermore, given that CD13^+^CD15^−^ basophiles were significantly more abundant than the control but that no significant difference was observed in the CD235a^+^ erythroid-related fraction, the biological effects of carbon-ion beams on hematopoiesis are very different from those of X-rays. These results suggest that a change in the cell fraction cannot be used to predict hematopoietic radiation injury with respect to dose dependency (Table 4), although it certainly produced radiation type-specific signal. Gorczyca *et al*. reported that basophils are positive for CD13, CD45, and CD123 (IL-3 receptor) and negative for CD15. In addition, they reported that late monocytes become positive for CD14 and the expression of CD14 is strongest by mature monocytes [17]. In the present study, we analyzed the co-expression of immunophenotypic pattern of myeloid populations such as CD13, CD14, and CD15 antigens. The results showed that CD13^+^CD14^+^CD15^+/−^ populations contained basophils and relative mature monocytes, whereas CD13^+^CD14^+/−^CD15^+^ populations contained mature basophils (Table 3), suggesting the myeloid differentiation pathway derived from HSPCs. We have reported that the residual HSPCs exposed to carbon-ion beams showed the upregulation tendency of IL3RA mRNA (code of IL-3 receptor antigen) [14]. Arock *et al*. reported that IL-3 is well known as the main growth and differentiation factor for basophils; thus, IL-3 supports the maturation of hematopoietic progenitors into basophils *in vitro* and *in vivo* [28]. It is suggested that basophil differentiation is readily induced in a cellular environment in which IL-3 is activated by IL3RA. Further analysis will be required to identify the correct difference. Subsequent analyses are under way to determine the mechanism(s) underlying the variation in cell surface antigen expression after exposure to X-rays or carbon-ion beam in myeloid hematopoiesis.

The main target in biological damage by ionizing radiation is genomic DNA, and cell death is induced when DNA damage exceeds repair capability [29], [30]. At this time, reactive oxygen species (ROS) produced by ionizing radiation directly induces DNA strand breaks and exerts various cytotoxic effects [31], [32]. In contrast, a previous report showed that ROS act as intracellular signaling molecules, such as in the mitogen-activated protein kinase (MAPK) pathway [33]. MAPK signaling has been demonstrated to play a key role in the maintenance of HSC quiescence [34]. In particular, the ERK MAPK is important, and the p38MAPK signaling pathway contributes to HSPC exhaustion in response to ROS-mediated oxidative stress. More precise approaches are required to elucidate the role of ROS in hematopoiesis derived from HSPCs exposed to carbon-ion beams and X-rays.

In conclusion, the present results suggest that carbon-ion beams inflict severe damage on the clonal growth of myeloid HSPCs and a significant difference in each cell fraction was dependent on the type of radiation, although the intensity of cell surface antigen expression by mature myeloid cells derived from HSPCs exposed to each type of radiation was similar to that by non-irradiated controls. This assessment of differences in LET is the first report describing radiation effects on human bone marrow hematopoietic lineages. The findings obtained in the present study shed light on the cellular and molecular mechanisms of ionizing radiation-induced myelosuppression and may lead to the development of novel strategies in radiation emergency medicine for space missions and nuclear accidents.

## Materials and Methods

### Growth factors and fluorescent antibodies

Recombinant human IL-3, SCF, and granulocyte/macrophage-colony stimulating factor (GM-CSF) were purchased from PeproTech, Inc. (Rocky Hill, NJ, USA). Erythropoietin (EPO) was purchased from Kyowa Hakko–Kirin (Tokyo, Japan), and granulocyte-colony stimulating factor (G-CSF) was purchased from Chugai Pharmaceutical Co. Ltd (Tokyo, Japan). These factors were administered at the following concentrations: IL-3, 100 ng/ml; SCF, 100 ng/ml; EPO, 4 U/ml; G-CSF, 10 ng/ml; GM-CSF, 10 ng/ml medium. Fluorescence-labeled fluorescein isothiocyanate (FITC)-conjugated anti-human CD34 monoclonal antibodies (mAbs), FITC-conjugated anti-human CD13 mAbs, FITC-conjugated anti-human CD235a mAbs, phycoerythrin (PE)-conjugated anti-human CD38 mAbs, PE-conjugated anti-human CD14 mAbs, PE-cyanin-5-forochrome tandem (PC5)-conjugated anti-human CD45 mAbs, and PC5-conjugated anti-human CD15 mAbs were purchased from Beckman Coulter Immunotech (Marseille, France). Mouse IgG_2a_-FITC, IgG_1_-FITC, IgG_1_-PE, and IgG_1_-PC5 (Beckman Coulter Immunotech) were used as isotype controls. The hemocyte marker was chosen to represent more than 1% of cell fractions in total MNCs.

### Collection and purification of placental/umbilical cord blood (CB) CD34^+^ cells

This study was approved by the Committee of Medical Ethics of Hirosaki University Graduate School of Medicine (Hirosaki, Japan). After informed consent was obtained from mothers following full-term deliveries by description and verbal explanation, CB was collected into sterile collection bags containing citrate phosphate dextrose anticoagulant (CBC-20; Nipro, Co., Osaka, Japan) until the flow ceased. These samples were separately isolated and used for each experiment. Within 24 h of collection, light-density mononuclear CB cells were separated by centrifugation on Limphosepar I (1.077 g/ml, Immuno-Biological Laboratories, Takasaki, Japan) for 30 min at 300×*g* and washed thrice with phosphate-buffered saline (PBS) containing 5 mM ethylenediaminetetraacetic acid (EDTA). These cells were then processed for CD34^+^ cell enrichment according to the manufacturer's instructions. An auto-MACS human CD34 selection kit (Miltenyi Biotech, Bergisch-Gladbach, Germany) was used for positive selection of the CD34^+^ cells. This isolated CD34^+^-enriched cell population is referred to as HSPCs in this study.

### Flow cytometry analysis

The expression of specific cell surface antigens was evaluated by direct immunofluorescence flow cytometry (FACSCaliber™; Becton Dickinson, Franklin Lakes, NJ, USA) using triple staining combinations of mAbs. In brief, the cells were incubated with saturated concentrations of the relevant mAbs for 20 min at room temperature, washed, and subjected to flow cytometry. For each experiment an isotype-matched irrelevant control mAb was used as a negative control.

### Irradiation

The CB CD34^+^ cells were exposed to each type of radiation. Monoenergetic carbon-ion beams (290 MeV/nucleon, spread-out Bragg peak: 60 mm, φ = 10 cm) were generated with an accelerator (Heavy Ion Medical Accelerator in Chiba) at the National Institute of Radiological Sciences (Chiba, Japan) [35]. In brief, a combination of wobbler magnets and a scatterer was used to obtain a uniform irradiation field. A range shifter made of Lucite disks was inserted immediately upstream of the samples to decrease the energy and to modulate LET at the sample position. In acrylic irradiation chambers of 1-mm thickness, LET ranged from 60 to 72 when the energy was adjusted to obtain 50 keV/µm at the sample center. The dose and dose rate were monitored during irradiation with a parallel-plate ionization chamber placed upstream of the range sifter. Before irradiation of the cell samples, this monitoring ionization chamber was calibrated with a standard ionization chamber placed at the sample position and calibrated to the national standard. LETs of the beams were calculated by fitting the measured residual range to the theoretical relationship between the depth and LET.

X-ray irradiation (150 kVp, 20 mA, 0.5-mm aluminum and 0.3-mm copper filters) was performed using an X-ray generator (MBR-1520R-3, Hitachi Medical Co. Ltd., Tokyo, Japan) at a distance of 45 cm between the focus and the target. The CD34^+^ cells were irradiated in serum-free medium. The dose was monitored with a thimble ionization chamber placed next to the sample during the irradiation. The dose rate was approximately 1 Gy/min.

### Liquid culture

CB CD34^+^ cells (0.5–2.0×10^3^ cells/ml in serum-free medium; a total volume 0.5 ml/well) were plated on 24-well plates and cultured in serum-free IMDM (Life Technologies Inc., Carlsbad, CA, USA) supplemented with BIT9500 (a serum substitute for serum-free culture, StemCell Technologies, Vancouver, Canada). The cell culture medium included cytokine combinations of five growth factors (IL-3, SCF, EPO, G-CSF and GM-CSF: 5GFs). This combination is optimal for proliferation and terminal maturation in the myeloid differentiation pathway [36]. The cultures were incubated at 37°C in a humidified atmosphere containing 95% air/5% CO_2_. The number of viable cells was counted on day 14 using the trypan blue dye exclusion method (Sigma-Aldrich Co. Ltd., St. Louis, MO, USA).

### Methylcellulose culture

We evaluated the radiosensitivity of lineage-committed myeloid hematopoietic progenitor cells, CFCs, in CD34^+^ cells prepared from individual samples of human CB. CFCs were composed of CFU-GM, BFU-E, and CFU-Mix. They were assayed using a methylcellulose culture as described previously [36]. The CD34^+^ cells (5.0×10^2^) were suspended in 1 ml of methylcellulose medium (MethoCult H4230, Stem Cell Technologies Inc.) supplemented with 5GFs. This mixture was transferred onto 24-well cell culture plates at 0.3 ml/well and then incubated at 37°C for 14 days in a humidified atmosphere containing 95% air/5% CO_2_. Colonies consisting more than 50 cells were counted using an inversion microscope. Each colony was identified based on its morphology according to the method described by Kaufman *et al*. [37]. In brief, a CFU-GM-derived colony is composed of granulocytes and macrophages and shows no color. In contrast, a BFU-E-derived colony is composed of erythroid cells containing hemoglobin and has a red color. Because CFU-Mix multilineage colonies contain many granulocytes, erythrocytes, macrophages, and megakaryocytes, red areas are visible in these colonies. The mature cells in each colony were checked using a cytospin smear technique described previously [38], [39].

### Statistical analysis

Statistical analysis was performed using the Origin software package (OriginLab® Pro version 8.5, Northampton, MA, USA) and SPSS version 17.0 (IBM, Chicago, IL, USA) for Windows. Dose-survival curves were fitted by the Levenberg–Marquardt algorithm, which combines the Gauss–Newton and steepest-descent methods, non-linear models based on the equation *y* = 1−(1−*exp*(−*x*/*D_0_*))^n^, and the values for *D_0_* (37% survival dose) and *n* (number of targets) were determined using a single-hit multitarget equation. Data were obtained from 4–8 independent experiments and two replications, and were compared using the Tukey–Kramer test, linear regression analysis, and two-factor factorial ANOVA. A *P* value of <0.05 was considered to be statistically significant.
